# LSD1 Promotes Bladder Cancer Progression by Upregulating LEF1 and Enhancing EMT

**DOI:** 10.3389/fonc.2020.01234

**Published:** 2020-07-28

**Authors:** Qiubo Xie, Tang Tang, Jian Pang, Jing Xu, Xingxia Yang, Linang Wang, Yiqiang Huang, Zhuowei Huang, Gaolei Liu, Dali Tong, Yao Zhang, Luofu Wang, Dianzheng Zhang, Weihua Lan, Qiuli Liu, Jun Jiang

**Affiliations:** ^1^Department of Urology, Daping Hospital, Army Medical University, Chongqing, China; ^2^Department of Bio-Medical Sciences, Philadelphia College of Osteopathic Medicine, Philadelphia, PA, United States

**Keywords:** bladder cancer, EMT, LSD1, LEF1, β-catenin

## Abstract

Epithelial-to-mesenchymal transition (EMT) is one of the important underlying molecular mechanisms for most types of cancers including bladder cancer. The precise underlying molecular mechanism in EMT-mediated bladder cancer progression is far from completed. LSD1, a histone lysine-specific demethylase, is known to promote cancer cell proliferation, metastasis, and chemoresistance. We found in this study that LSD1 is highly upregulated in bladder cancer specimens, especially those underwent chemotherapy, and the elevated levels of LSD1 are highly associated with bladder cancer grades, metastasis status, and prognosis. Inhibiting or knockdown LSD1 repressed not only EMT process but also cancer progression. Mechanistically, LSD1 complexes with β-catenin to transcriptionally upregulate LEF1 and subsequently enhances EMT-mediated cancer progression. More importantly, LSD1 specific inhibitor GSK2879552 is capable of repressing tumor progression in patient-derived tumor xenograft. These findings altogether suggest that LSD1 can serve as not only a prognostic biomarker but also a promising therapeutic target in bladder cancer treatment.

## Introduction

Bladder cancer (BCa) is one of the most frequently diagnosed cancers worldwide with about 429,800 new cases and 165,100 deaths annually (1). Epithelial-mesenchymal transition (EMT) plays an important role in recurrence, chemoresistance, and progression of BCa (2) with increased EMT transcription factors (EMT-TFs) including Snail, Zeb-1, Twist and Slug, and altered expression of factors involved in cell–cell interaction such as E-cadherin, N-cadherin, and vimentin. Multiple lines of evidence indicate that epigenetic regulation play an important role in BCa cell EMT (3). A better understanding of the underlying epigenetic mechanisms of EMT in BCa will not only enhance our understanding of BCa progression but also provide potential therapeutic strategies.

Lysine-specific demethylase 1 (LSD1; also known as AOF2 or KDM1a) can function as either a transcriptional activator or a transcriptional repressor. By demethylating histone 3 lysine 4 (H3K4), LSD1 represses gene expression transcriptionally (4, 5). On the other hand, LSD1-mediated demethylation of H3K9 promotes transcriptional activation (6). Therefore, LSD1 can simultaneously up- and down-regulate the expression of a wide spectrum of genes involved in a variety of biologic processes such as proliferation, stem cell pluripotency, embryonic development, and EMT (7–11). In the cancers of the prostate (12), lung (13), ovarian (14), and colon (15), LSD1 serves as a key positive regulator of EMT and associate with poor prognosis. Our previous studies found that elevated levels of LSD1 were highly associated with the grades of BCa (16). However, the role of LSD1 in EMT and BCa progression is largely unknown.

In this study, we demonstrated that LSD1 promotes BCa cell EMT by upregulating LEF1. Based on the fact that the levels of LSD1 are highly associated with cancer grades, metastasis status, prognosis, and inhibition of LSD1 significantly repressed BCa cell proliferation, we propose that the levels of LSD1 can serve as a prognostic biomarker and targeting LSD1 could be a promising therapeutic strategy for BCa treatment.

## Materials and Methods

### Patients and Bladder Cancer Specimens

All patient samples were collected by the Department of Pathology with approval from the Research Ethics Committee of Daping Hospital, Army Military Medical University and written informed consent was obtained from each patient. All procedures involving human participants were carried out in accordance with the ethical standards of the institutional research committee and with the 1964 Helsinki Declaration and its later amendments or comparable ethical standards. A total of 122 formalin-fixed, paraffin-embedded samples from bladder cancer patients were collected and used for tissue microarray (TMA) in this study. Among them, 15 samples were excluded because no tumor cells were identified. Therefore, 107 specimens were further analyzed, including 24 low-grade non-muscle-invasive bladder cancer (NMIBC), 68 high-grade muscle-invasive bladder cancer (MIBC), and 15 Lymph node (LN) metastatic tissue samples from high-grade MIBC patients. In addition, 12 pairs of BCa tissues and pair-matched histologically normal tissues were collected for WB. Another 20 pairs of BCa tissue specimens before and after intravesical instillation or neoadjuvant therapy were collected for IHC. All the clinical information of the patients was supplied in Supplementary Tables S1–S3.

### Animal Studies

All nude mice and severe combined immune deficiency NPG/Vst mice with an average age of 6–8 weeks were purchased and bred by the Division of Laboratory Animal Medicine at Army Medical University. All work performed on animals were in accordance with international laws (EEC Council Directive 86/609, O.J. L 358. 1, December 12, 1987; Guide for the Care and Use of Laboratory Animals, United States National Research Council, 1996) and has been approved by the Institutional Animal Care and Use Committee at Army Medical University. Animals were fed with standard laboratory chow with free access to tap water. To establish the xenograft models, 2 × 10^6^ T24 cells with or without LSD1 knockdown in 0.1 ml culture medium were mixed with Matrigel (BD Biosciences, NO.356234) at a ratio of 1:1, and injected subcutaneously into the flank of the nude mice. The injections were carried out in a sterilized hood. A first-generation patient-derived xenograft (PDX) of metastatic bladder cancer tissue was established in the NPG mice. The mice were treated with either vehicle or GSK2879552 (Selleck, 5 mg/kg) for 4 weeks with the sizes of the tumors monitored every other day. Tumor volume was measured with a Vernier caliper. Then, the mice were sacrificed and the tumor tissues were harvested and fixed in 10% formaldehyde solution and embedded in paraffin for histology analyses. For the tail vein injection model, the nude mice were intravenously injected in the tail vein with T24 cells with or without LSD1 knockdown (3 × 10^5^) per mouse. After 2 weeks, the animals were euthanized, and the lungs were excised and embedded in paraffin.

### IHC Staining

The paraffin-embedded tissues were sectioned at 4 mm thickness and arranged on glass slides in sequence. In brief, slides were baked at 60°C for 6 h, followed by deparaffinization with xylene, rehydrating in graded ethanol, and 3% hydrogen peroxide to block the endogenous peroxidase activity. The sections were submerged in citrate or EDTA buffer and microwaved for antigen retrieval. Goat serum (ZSGB-BIO, China) was used to block nonspecific background and then the sections were incubated at 4°C with specific primary antibodies against LSD1 (1:100 Abcam), E-cadherin (1:500 Abcam), N-cadherin (1:200 Proteintech), Vimentin (1:1,000 Proteintech), Twist (1:200 Abcam), followed by the secondary antibody conjugated with streptavidin-biotin-horseradish peroxidase complex (Biotinylated Anti-Rabbit IgG, SP-9001; Biotinylated Anti-Mouse IgG, SP-9002, ZSGB-BIO, China). The slides were scanned by using a computerized image system composed of an Olympus CCD camera (Tokyo, Japan) connected to a Nikon eclipse Ti-s microscope (Tokyo, Japan) and captured by NIS-Elements F3.2. All of the slides were assessed by two urological pathologists.

### Cell Culture

Human bladder cancer cell lines (T24 and BIU87) were obtained from Cell Bank of Shanghai Institutes for Biological Sciences (Chinese Academy of Sciences), and cultured in RPMI 1640, supplemented with 10% fetal bovine serum (Life Technologies, Carlsbad, CA), 100 U/ml penicillin and 100 mg/ml streptomycin (Invitrogen) and cultured in a humidified incubator at 37°C and 5% CO_2_.

### Lentivirus Production and Knockdown of LSD1 by siRNA

For LSD1 knockdown experiments, T24 and BIU87 cells were transfected with lentiviruses Lv-shRNA-LSD1 and Lv-scramble (SBO Medical Biotechnology, Shanghai, China). Targeting sequences for shLSD1: CCACCTGACAGTAAGGAAT. According to the manufacturer's instructions, the LSD1-specific shRNA-expressing or control lentiviruses were incubated together with a lentiviral transfection enhancer (Polybrene 5 mg/mL, Sigma–Aldrich). T24 and BIU87 cells with LSD1 knockdown were established by screening with puromycin. In addition, LSD1 was knocked down in T24 and BIU87 cells by scrambled or specific siRNAs. Targeting sequences for siRNA1 of LSD1: GCTCGACAGTTACAAAGTT, siRNA2 of LSD1: GTTGGATAATCCAAAGATT. siRNAs of LSD1 or β-catenin were transfected into T24 or BIU87 cells using Lipo3000 (Invitrogen) following the manufacturer's instructions.

### Cell Viability Assay

Cells were seeded into 96-well-plates and after grown to 60–75% confluence in RPMI 1640 with 10% FBS for 48 h. The viabilities of the cells were evaluated by using the Cell Counting Kit-8 (CCK8) assay. Briefly, at the end of the exposure, cells were aspirated, and rinsed with PBS then treated with 10 μl/well CCK-8 for 2 h at 37°C. Absorbance was measured at 450 nm spectrophotometrically (Bio-Rad, Hercules, CA, USA).

### Wound Scratch Assay

Cell migration was monitored in a wound scratch assay. Briefly, bladder cancer cells were seeded to a 6-well-plate at a density of 2 × 10^5^ cells/well and cultured in the medium for 24 h. A scratch was made with a sterile 10 ml pipette tip in a confluent cell monolayer, washed with PBS, and incubated in a serum-free medium. Images of the scratches were captured at the beginning of the experiment and after 24 h with an inverted microscope (CK40 F200; Olympus, Japan).

### Transwell Assays

To assess cell migration and invasion *in vitro*, we used 24-well transwell chambers with or without Matrigel. Cells were trypsinized and seeded into the top chamber at a density of 5 × 10^4^ cells per well in 200 ml Dulbecco's modified Eagle's medium. The outer chambers contained 800 ml of medium (10% fetal calf serum). After incubation at 37°C for 24 h, cells attached to the upper surface of the membrane were carefully removed with cotton swabs, whereas cells that reached the underside of the chamber were fixed with 10% formalin and stained with crystal violet for 3 min at room temperature and counted.

### Western Blotting and Immunoprecipitation

For Western blotting, cells were seeded in 6-well-plates 2 × 10^5^ cells/well. Cell lysates were separated on SDS-PAGE followed by western blotting assay with antibodies against LSD1 (1:1,000 Abcam), LEF1 (1:1,000 Abcam), Twist (1:500 Abcam), E-cadherin (1:2,000 Abcam), N-cadherin (1:500 Proteintech), Vimentin (1:2,000 Proteintech), and β-actin (1:5,000 Cell Signaling). IP was performed according to the protocol. Briefly, after cold PBS washing, 2 × 10^7^ cells were scraped into lysis buffer containing protease inhibitors for 30 min on ice, then sample lysis was centrifuged at 14,000 × g for 20 min at 4°C. The supernatants were incubated with normal IgG or indicated antibodies and protein A/G-beads. The precipitated complexes were washed with lysis buffer and boiled for 5 min in SDS sample buffer. The antibodies used for this process are as follows: LSD1 (1:1,000 Abcam), β-catenin (1:1,000 Abcam), and anti-mouse or -rabbit IgG (Pierce) was used as a secondary antibody.

### RNA Extraction, Quantitative Real-Time PCR

Total RNA samples from cultured cells were extracted using Trizol reagent (Invitrogen) according to the manufacturer's instructions. Quantitative real-time PCR (qRT-PCR) was performed by real-time PCR (BioRad, Hercules, CA, USA). The fold change between target gene mRNA transcripts and control glyceraldehyde-3 phosphate dehydrogenase (GAPDH) were calculated and shown in the histogram. Primer sequences can be found in Supplementary Table S4. All the experiments were performed in triplicate.

### Immunofluorescence Assay

A total of 5 × 10^4^ Cells was seeded in the confocal dish. After 2 days of culture, the cells were washed with phosphate buffer saline (PBS), fixed in 4% paraformaldehyde, and cell membranes were permeabilized with 0.3% Triton X-100 solution. Next, cells were blocked with 5% bovine serum albumin (BSA) in 1% Triton X-100 and then blotted at 4°C overnight with different primary antibodies against LEF1 (1:200 CST), β-catenin (1:200 Abcam). Fluorescence-conjugated secondary antibodies (Alexa Fluor antibody, Life Technologies) were then added to these dishes. Cells were further washed and the nuclei were stained with 2-(4-Amidinophenyl)-6-indolecarbamidine dihydrochloride (DAPI) (Sigma). These confocal dishes were visualized using a Carl Zeiss confocal fluorescence microscope (LSM 780).

### Chromatin-Immunoprecipitation Assays and RT-PCR

The CHIP assays were performed using a magnetic CHIP kit (Active Motif). The detailed procedure was as described in the kit provided by the manufacturer. Briefly, cells were fixed by 1% formaldehyde, fragmented by enzymatic shearing. LSD1, β-catenin, LEF1 antibodies from Abcam were then used for immunoprecipitation. After washing and reverse-crosslinking, the precipitated DNA was purified and amplified by real-time-PCR machine. Quantitative real-time polymerase chain reaction (RT–PCR) was performed by using the SYBR Green Master Mix (Invitrogen) based on iCycle System (Bio-Rad). PCR data were analyzed using Graph Pad Prism (Graph Pad Software, San Diego, California, USA).

### Statistical Analysis

GraphPad Prism 5.0 (Graph Pad Software, San Diego, California, USA) was used for all statistical analyses. Data were presented as means ± standard deviation (SD). If the data follow a normal distribution, the statistical significance of differences between two groups of data was analyzed by *t*-test and χ^2^-test, differences among several groups were analyzed by one-way analysis of variance followed by the Least Significant Difference (LSD) procedure for comparison of means. For those do not fit a normal distribution, non-parametric statistical tests were used. A *P*-value of <0.05 was considered statistically significant.

## Results

### Elevated Levels of LSD1 in BCa Are Correlated With Poor Prognosis

**Figure 1:**
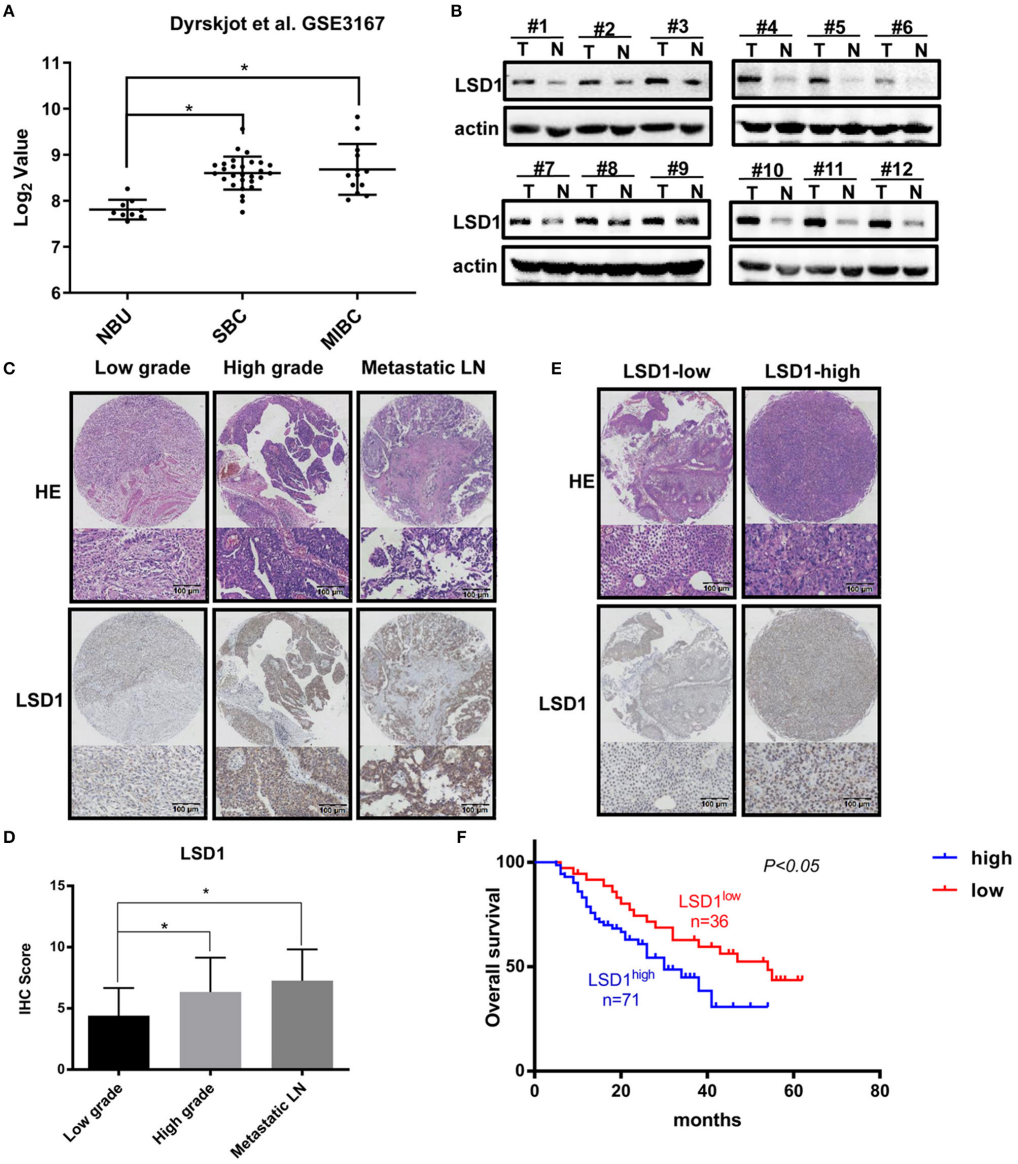
Elevated levels of LSD1 in bladder cancer are correlated with poor prognosis. **(A)** Datasets from Gene Expression Omnibus (GEO) showed that LSD1 was relatively highly expressed in BCs tissues than normal urothelium. **(B)** 12 pairs of BCa tissues and pair-matched histologically normal tissues were estimated by WB. **(C,D)** Representative H&E staining and IHC results for LSD1 in the TMA from bladder cancer patients. **(E,F)** The overall survival of 107 patients with LSD1^high^ or LSD1^low^ BCa was analyzed by the log-rank test. *P < 0.05.

Elevated levels of LSD1 in a wide spectrum of malignancies including prostate cancer (12), colon cancer (17), lung cancer (18), liver cancer (19), and thyroid carcinoma (20) are highly correlated with lymph node metastasis and poor prognosis. Based on our finding that elevated levels of LSD1 were highly related to the grades of the bladder cancers (16), we hypothesized that LSD1 plays an oncogenic role in BCa development and/or progression. To test our hypothesis, we first analyzed the publicly available datasets (submitted by Dyrskjøt et al., E-mail: lars@clin.au.dk, available at: https://www.ncbi.nlm.nih.gov/geo/query/acc.cgi?acc5GSE3167) in the Gene Expression Omnibus (GEO) and found that compared to that in the normal urothelium the levels of LSD1 are significantly higher in both NMIBC and MIBC (Figure 1A). We then compared the levels of LSD1 in BCa specimens with their surrounding normal tissues from a cohort of 12 BCa patients. The levels of LSD1 in tumor tissues are consistently higher than that of their corresponding normal tissues albeit LSD1 expression varies greatly among patients (Figure 1B). Next, we conducted immunohistochemistry (IHC) staining against LSD1 on TMA in patients with low-grade NMIBC, high-grade MIBC, and metastatic MIBC (Figure 1C). Figure 1D showed that the level of LSD1 is significantly elevated in high-grade and metastatic cancers. More importantly, patients with elevated levels of LSD1 in their BCa (Figure 1E) also have shortened overall survival (Figure 1F). These results altogether indicated that LSD1 is associated with BCa progression and poor prognosis.

### The Role of LSD1 in BCa Cell Migration, Invasion, and Proliferation

**Figure 2:**
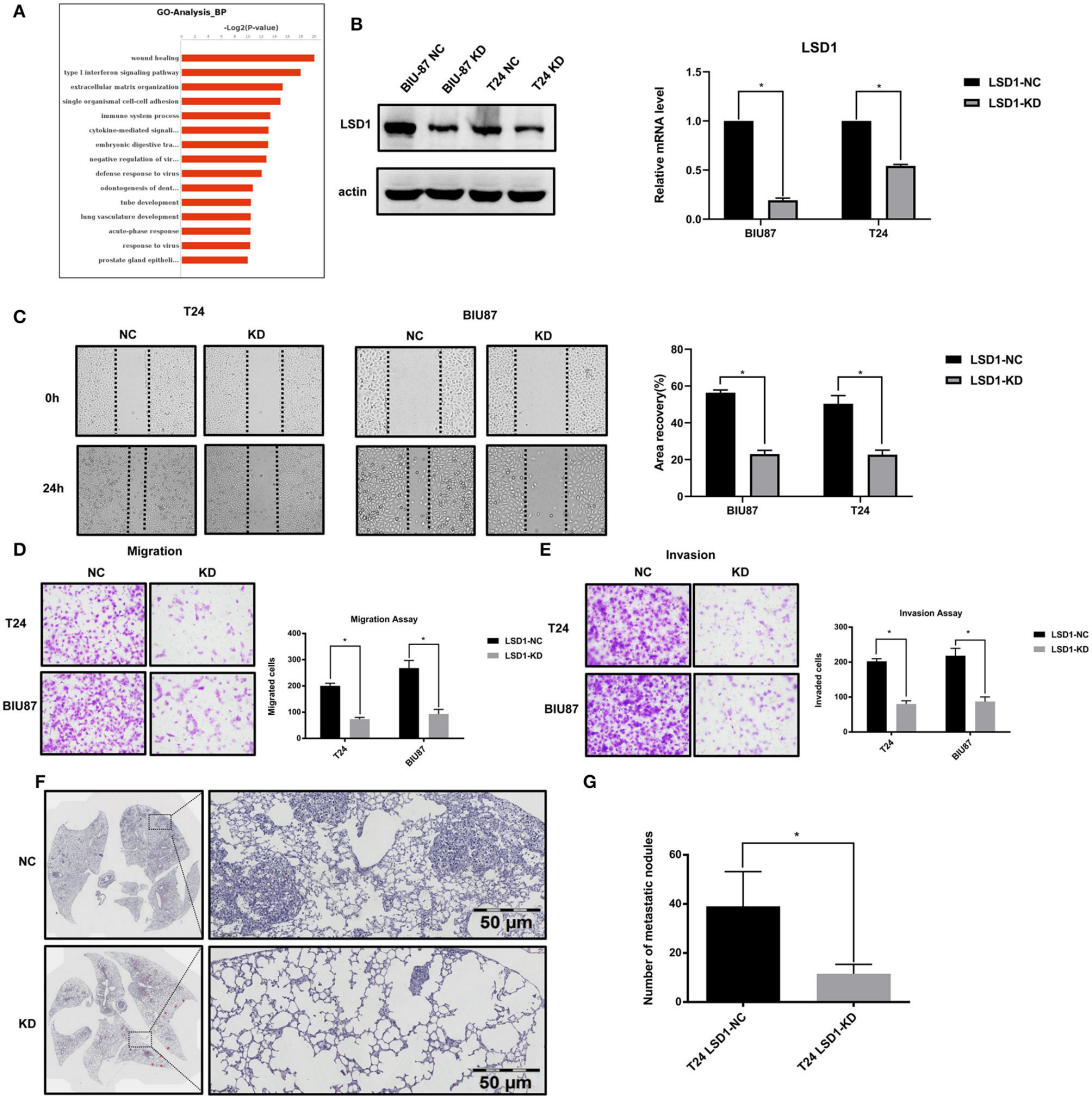
LSD1 promotes the migration, invasion, and proliferation of bladder cancer cell. **(A)** The top 15 GO categories of biological process (BP) of RNA-Seq result. **(B)** Expression of LSD1 analyzed by Western blotting and RT-qPCR after transfection with shRNA for LSD1 in T24 and BIU87 cells. **(C)** Cells were seeded in 6-well-plates in growth media and cultured for 24 h. Cell migration was estimated by scratch-wound healing assay. **(D,E)** T24 cells with or without LSD1 knockdown were seeded in 24-well transwell chambers with or without Matrigel in growth media and cultured for 48 h. Cell migration **(D)** and invasion **(E)** were estimated. **(F,G)** H&E stained section of lung showing metastatic lesions collected 14 days after the injection of T24 cells with or without LSD1 knockdown into the tail vein of nude mice. A significant reduction in lung metastasis between the two cohorts can be observed. Quantifications were shown on right **(G)**. *P < 0.05.

Given that LSD1 can promote cell invasion and migration in many cancer cells, we decided to exam if LSD1 has the same effect in BCa cells. To do so, we first knocked down LSD1 in both T24 and BIU87 cells by shRNAs specifically against LSD1 and conducted RNA-Seq to identify differentially expressed genes (DEGs) in the cells with or without LSD1 knockdown. Among the 928 DEGs, 504 and 424 were up- and downregulated, respectively. The DEGs were then subjected to gene ontology (GO) category and KEGG pathway analyses. The top 15 GO categories of biological processes (BP) were related with wound healing (Figure 2A), a process closely relevant to EMT. Results from scratch assay showed that wound scratch closed up much slowly when LSD1 is knocked down (Figures 2B,C), and these findings are consistent with that from transwell assays (Figures 2D,E). Next, we injected T24 cells with or without LSD1 knockdown to the tail-vein of mice. Fourteen days post-injection, mice were sacrificed and the number of nodules in the lungs which represent the invasive capacity was estimated. Figures 2F,G showed that knockdown LSD1 reduced the number of lung metastasis nodules significantly. Results from CCK-8 assays showed that the proliferation of both T24 and BIU87 cells was also repressed when LSD1 is knocked down (Supplementary Figures S1A,B). Moreover, when cells were injected into the flanks of nude mice to form xenograft tumors, the weight and size of xenografts derived from T24 with or without LSD1 knockdown demonstrated the indispensability of LSD1 in BCa (Supplementary Figures S1C–E). These data altogether suggested that LSD1 plays an important role in BCa cell migration, invasion and proliferation.

### LSD1 Regulates BCa EMT *in vitro* and *in vivo*

**Figure 3:**
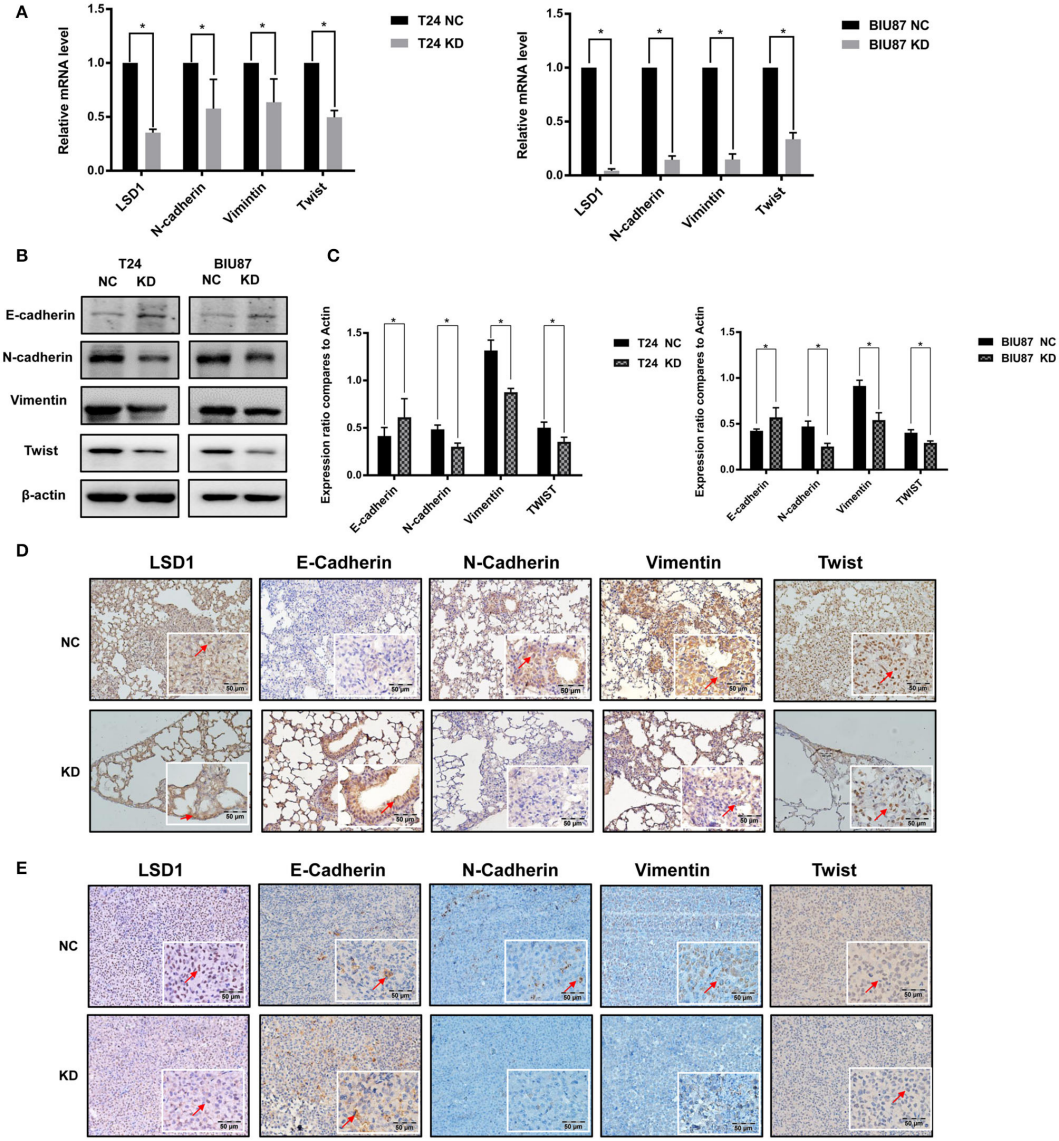
LSD1 regulates BCa EMT *in vivo* and i*n vitro*. **(A–C)** T24 cells with or without LSD1 knockdown were seeded in 6-well-plates and cultured for 48 h; mRNA and protein levels of EMT markers E-Cadherin, N-Cadherin, Vimentin, and Twist were determined by quantitative RT-PCR and Western blotting. The data represent means ± S.D. *P < 0.05. **(D)** Lungs collected from T24 cells with or without LSD1 knockdown tail vein injected model of nude mice were conducted IHC staining with antibodies against E-cadherin, N-cadherin, Vimentin and Twist. **(E)** Tumors collected from T24 cells with or without LSD1 knockdown subcutaneous xenograft model of nude mice treated as indicated were conducted IHC staining with antibodies against E-cadherin, N-cadherin, Vimentin, and Twist.

Given that (1) EMT plays important roles in BCa progression (2); and (2) treatments such as cisplatin, doxorubicin (21), and mitomycin C (22) can enhance BCa cell EMT process, we decided to explore the role of LSD1 in BCa cell EMT. Results from q-PCR and WB showed that knockdown LSD1 resulted in a significant reduction of both mRNA (Figure 3A) and protein (Figures 3B,C) levels of the mesenchymal markers N-cadherin, Vimentin, and Twist with elevated levels of the epithelial marker E-cadherin. Meanwhile, upregulated N-cadherin, Vimentin, and Twist and downregulated E-cadherin were seen in tumors formed in the lungs (Figure 3D). These findings were further substantiated by the IHC results of the xenografts derived from T24 with or without LSD1 knockdown (Figure 3E). All these results suggested that LSD1 plays an important role in BCa cell EMT *in vivo*. Then, we conducted IHC staining of BCa specimens collected from 20 patients before and after chemotherapy. The 10 patients with NMIBC were treated with local intravesical instillation and Supplementary Figure S2A showed that local intravesical instillation with gemcitabine induced BCa cell EMT evidenced by up-regulated mesenchymal markers (N-cadherin, Vimentin, and Twist) and down-regulated epithelium marker E-cadherin. Of note, elevated levels of LSD1 were seen in samples post local intravesical instillation. Another 10 patients with MIBC were treated with neo-adjuvant systemic chemotherapy and Supplementary Figure S2B showed that systemic chemotherapy also resulted in increased mesenchymal phenotype and reduced epithelial phenotype evidenced by upregulated mesenchymal markers (N-cadherin, Vimentin, and Twist) and downregulated epithelium marker E-cadherin. More importantly, LSD1 is also upregulated by systemic chemotherapy. These results suggested that either local intravesical installation or systemic chemotherapy can induce BCa cell EMT and LSD1 plays an indispensable role in this process.

### LSD1 Regulates LEF1 by Forming a Complex With β-Catenin

**Figure 4:**
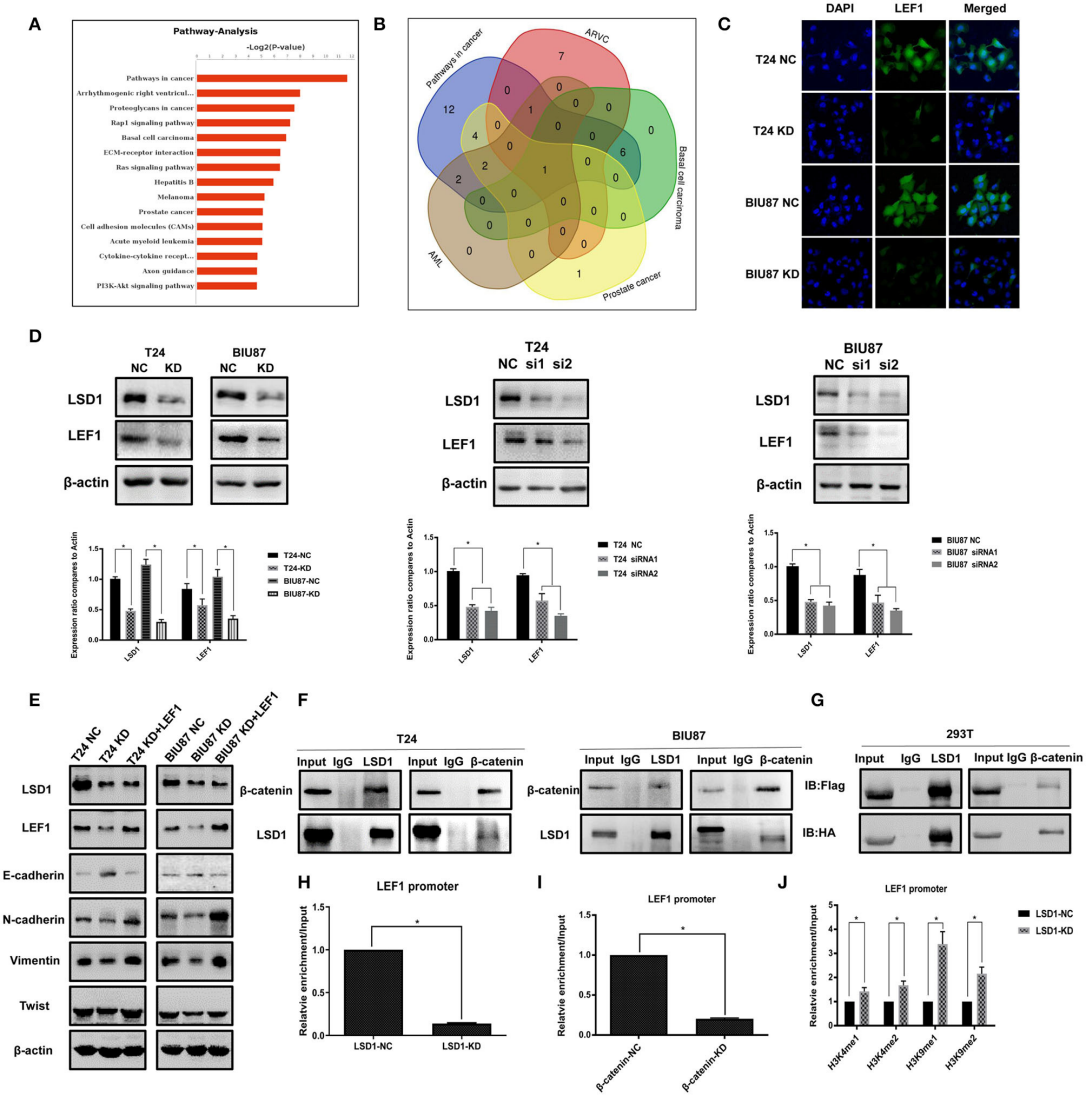
LSD1 regulates LEF1 by forming a complex with β-catenin. **(A)** The top 15 pathways of RNA-Seq result. **(B)** Venn diagram showed that the overlapping gene among the top five pathways was LEF1. **(C)** Confocal microscopy was used to determine the LEF1 protein expression in T24 cells with or without LSD1 knockdown. **(D)** Expression of LEF1 analyzed by RT-qPCR after transfection with shRNA or siRNAs for LSD1 in T24 and BIU87 cells. Expression of LEF1 analyzed by Western blotting after transfection with shRNA or siRNAs for LSD1 in T24 and BIU87 cells. **(E)** EMT markers were determined by Western blotting in T24 and BIU87 cells with LSD1 knockdown when the expression of LEF1 was rescued by transient transfection of a plasmid expressing LEF1. The data represent means ± S.D. *P < 0.05. **(F**) T24 and BIU87 cells were lysed and subjected to co-immunoprecipitation with LSD1 and β-catenin antibodies. **(G)** 293T cells were transfected with indicated plasmids. co-immunoprecipitation was conducted by using Flag and HA antibodies, immunoblot was analyzed with the above antibodies. **(H)** T24 cells with or without LSD1 knockdown were subjected to CHIP assays with LSD1 antibody. **(I)** T24 cells with or without β-catenin knockdown were subjected to CHIP assays with LSD1 antibody. **(J)** T24 cells with or without LSD1 knockdown were subjected to CHIP assays with H3K4me1, H3K4me2, H3K9me1, and H3K9me2 antibodies.

RNA-Seq showed that LSD1 is highly involved in both wound healing (Figure 2A) and cancer development (Figure 4A). Intriguingly, LEF1 appeared to be involved in all top five pathways as shown in the Venn diagram (Figure 4B). Given the important role of LEF1 in EMT and LSD1 is enriched at the transcriptional start site of the LEF1 gene in breast cancer (23), we focused our attention on LSD1-regulated LEF1 in BCa. To confirm the RNAseq results, we conducted immunofluorescence staining on T24 and BIU87 cells and found that LEF1 is much less expressed when LSD1 is knocked down (Figure 4C). Then, qPCR and WB were conducted on T24 and BIU87 cells when LSD1 is knocked down by two different siRNAs. Both the mRNA and the protein (Figure 4D) levels of LEF1 were significantly downregulated when LSD1 is knocked down. To determine the role of LEF1 in LSD1-enhanced BCa EMT, we overexpressed LEF1 by transient transfection of a plasmid expressing LEF1 in T24 and BIU87 cells with LSD1 knockdown. Figure 4E showed that LEF1 is successfully expressed in these cells and the overexpressed LEF1 is capable of rescuing the downregulated mesenchymal markers N-cadherin, Vimentin, and Twist and upregulated epithelial marker E-cadherin resulted from LSD1 knockdown, strongly suggesting the role of LSD1 in BCa EMT is LEF1-dependent. Given the fact that Wnt/β-catenin transcriptionally regulates LEF1 (24, 25) and LSD1 is enriched at the transcriptional start site of the LEF1 gene in breast cancer (23), we speculated that LSD1 may regulate LEF1 in BCa by cross-talking with β-catenin. We first conducted immunoprecipitation assays and showed that LSD1 and β-catenin can precipitate each other reciprocally in both T24 and BIU87 cells (Figure 4F). These results were further substantiated by immunoprecipitation assays with the lysate of 293T cells when Flag-tagged LSD1 and HA-tagged β-catenin were co-expressed (Figure 4G). More importantly, the recruitment of LSD1 on LEF1 promoter is significantly reduced when either LSD1 or β-catenin were knocked down (Figures 4H,I) suggesting the essentiality of β-catenin in the recruitment of LSD1. Since knockdown LSD1 has also remarkably increased the levels of H3K4me1, H3K4me2, H3K9me1, and H3K9me2 on the promoter region of LEF1 (Figure 4J), we conclude that LSD1's demethylase activity is indispensable for LEF1 upregulation. These data altogether demonstrated that in BCa cells LSD1 complexes with β-catenin and to upregulate LEF1 transcriptionally. Upregulated LEF1, in turn, mediates LSD-enhanced EMT by regulating the expression of specific epithelial and mesenchymal markers.

### Inhibiting LSD1 Attenuates BCa Cell Proliferation *in vivo*

**Figure 5:**
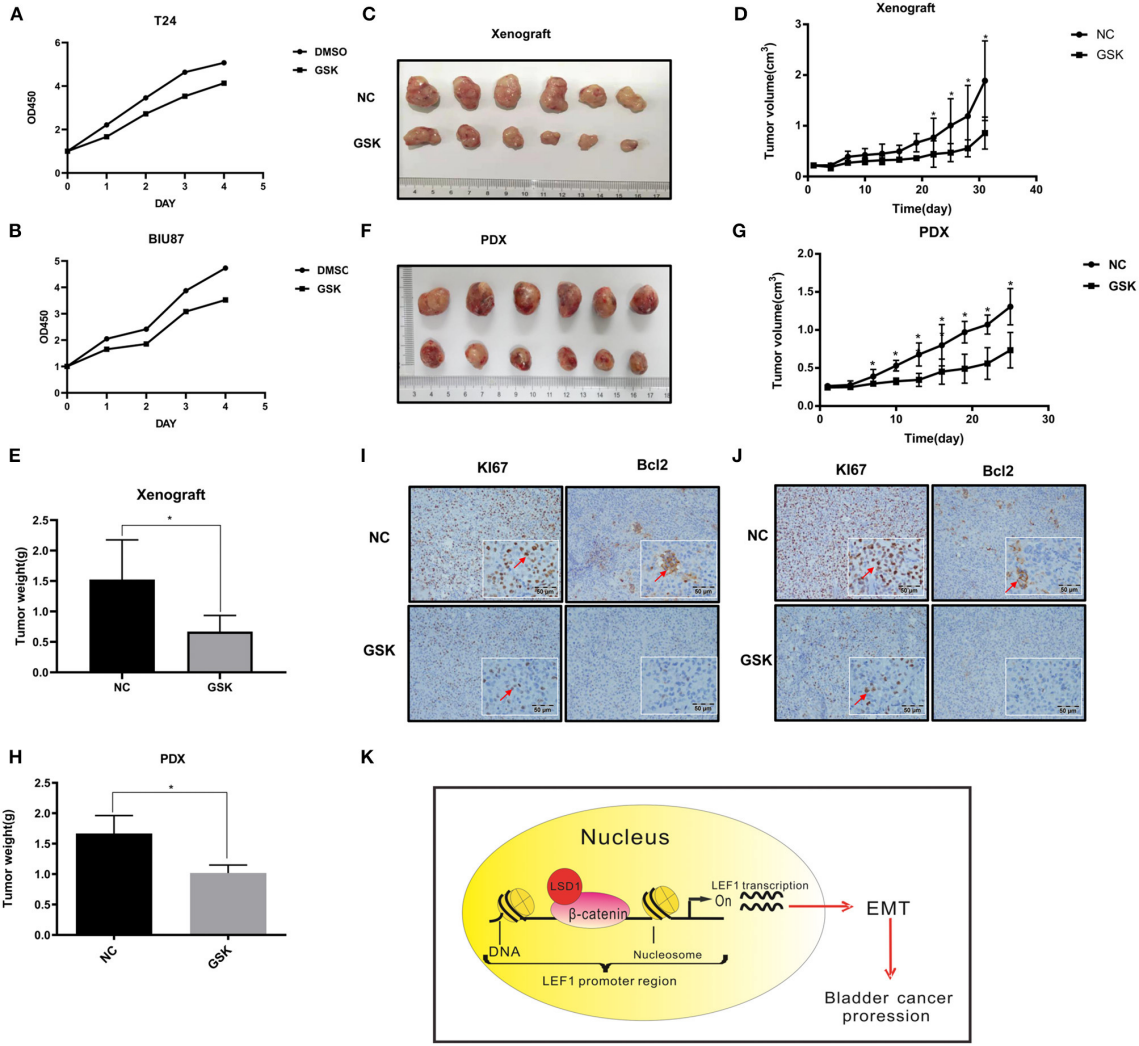
Inhibiting LSD1 attenuates BCa cell proliferation *in vivo*. **(A,B)** T24 and BIU87 cells with or without GSK were seeded in 96-well-plates with 1 × 10^3^ cells per well in growth media cultured for 7 days. Cell viabilities were estimated by CCK8 every other day. **(C–H)** Nude mice bearing T24 cell-derived or patient-derived xenograft (PDX) metastatic bladder cancer tissue was treated with or without GSK2879552 for 4 weeks; the tumors were measured and weighed. The data represent means ± S.D. *P < 0.05. **(I)** Xenografts collected were conducted IHC staining with antibodies against Ki67 (proliferation marker) and Bcl-2 (apoptosis marker). **(J)** Patient-derived xenograft (collected were conducted IHC staining with antibodies against Ki67 and Bcl-2. **(K)** Schematic model of the hypothesized mechanism by which LSD1 promotes EMT development.

It has been reported that LSD1 inhibitor GSK287955 can repress proliferation of both AML and small-cell lung cancer (SCLC) cells *in vitro* and the growth of NCI-H1417 SCLC xenograft *in vivo* (18, 26). We decided to test if GSK2879552 can inhibit LSD1-mediated BCa progression. Results from CCK-8 assays showed that proliferation of both T24 and BIU87 cells is inhibited by GSK2879552 (Figures 5A,B). Then, T24 cells were injected subcutaneously into nude mice and the tumors were allowed to grow for 10 days. The mice were treated with either vehicle or GSK2879552 (5 mg/kg) for 4 weeks with the sizes of the tumors monitored every other day. The tumors in mice treated with GSK2879552 are not only much smaller but also weigh much lesser (Figures 5C–E). Finally, we estimated the effect of GSK2879552 in the patient-derived xenograft (PDX) model using metastatic BCa tissues and found that GSK2879552 is capable of inhibiting the growth of PDX significantly (Figures 5F–H). Furthermore, the results of IHC showed the levels of Ki67 and Bcl-2 in xenografts derived from either cancer cells or PDX were also inhibited by GSK2879552 (Figures 5I,J). These data altogether suggest that inhibiting LSD1 by GSK2879552 could be a promising therapeutic strategy in BCa treatment.

## Discussion

BCa is one of the most frequently diagnosed cancers and its treatment remains a great challenge (27). Although it is well-accepted that EMT plays a crucial role in the progression of BCa and epigenetic regulation is important in EMT progress, the precise underlying mechanisms are largely unknown. We found in this research that LSD1 is capable of enhancing EMT process and BCa cancer progression by complexing with β-catenin to transcriptionally upregulate LEF1 (Figure 5K). We also demonstrated that targeting LSD1 by specific inhibitor GSK2879552 could be a new therapeutic strategy for BCa treatment.

EMT is involved in numerous biological and pathological processes including embryonic development, wound healing, cancer cell metastasis, and drug resistance (28). For cancer cells, loss of apical-basal polarity, cell-cell adhesion, and transit to invasive mesenchymal cells ultimately leads to intravasation (29). Poor prognosis is frequently accompanied by enhanced EMT in different cancers, including pancreatic cancer, breast cancer (30), and lung cancer (31, 32). It has been suggested that EMT is also a crucial process in BCa progression and metastasis (2). Non-muscle-invasive BCa carries a high risk of progressing to muscle-invasive BCa. This process is accompanied by decreased E-cadherin expression, suggesting that EMT is involved in the progression in BCa (10). We demonstrated that knockdown of LSD1 inhibited EMT process evidenced by increased protein levels of E-cadherin and decreased mRNA and protein levels of N-cadherin, Vimentin and Twist in tumor cell lines *in vitro* and xenograft *in vivo*. In addition, we showed that intravesical instillation and neoadjuvant therapy can upregulate LSD1 and EMT in BCa cells, and these findings are in accordance with that fact that EMT could be induced by treatments such as cisplatin, doxorubicin, and mitomycin C (21).

Epigenetic modifications play important roles in different diseases (33) and overexpression of LSD1 correlates with the invasion, migration, metastasis, and poor clinical outcome of different cancers (34–37), and EMT is closely correlated with the migration and invasion of various types of tumors (38). Although most studies show that LSD1 can enhance EMT, the underlying molecular mechanisms in BCa is unclear. In this study, we demonstrated that LSD1 is associated with BCa malignant potential progression and poor prognosis. We also propose that in BCa cells the LSD1-regulated EMT markers is LEF1-dependent because (1) knockdown LSD1 not only interrupted the expression of EMT markers but also downregulated LEF1, and (2) overexpressed LEF1 can rescue the expression of EMT markers in LSD1 knockdown cells. These findings are in line with the reports that LEF1 is highly involved in both tumorigenesis and EMT (39, 40). In addition, pharmacological inhibition or knockdown of LSD1 significantly decreased migration and invasion of cancers in the colon (15), prostate (12), lung (13), and ovarian (14). In this study, we demonstrated that GSK2879552 is capable of repressing tumor progression of both T24- and patient-derived xenograft. Since both preclinical and clinical data (18, 41) indicate that GSK2879552 is a strong antitumor reagent with acceptable tolerability and safety profile in patients with advanced cancers including small-cell lung cancer (SCLC) and AML, we propose that inhibiting LSD1 by GSK2879552 could be a potential therapeutic option for patients with BCa.

## Data Availability Statement

The datasets presented in this study can be found in online repositories. The names of the repository/repositories and accession number(s) can be found in the article/Supplementary Material.

## Ethics Statement

The studies involving human participants were reviewed and approved by The Research Ethics Committee of Daping Hospital, Army Military Medical University. The patients/participants provided their written informed consent to participate in this study. The animal study was reviewed and approved by The Institutional Animal Care and Use Committee at Army Medical University. Written informed consent was obtained from the individual(s) for the publication of any potentially identifiable images or data included in this article.

## Author Contributions

JJ, QL, and WL designed the study. QX and TT performed the main experiments. JP, JX, and XY performed animal experiments. LiW, YH, and ZH collected patient specimens. GL and DT acquired the data. YZ and LuW analyzed the data. QX wrote the manuscript. DZ revised the article manuscript. All authors have read and approved the final manuscript.

## Conflict of Interest

The authors declare that the research was conducted in the absence of any commercial or financial relationships that could be construed as a potential conflict of interest.
