# Supplementary material for: LSD1 Promotes Bladder Cancer Progression by Upregulating LEF1 and Enhancing EMT
Source: Front Oncol. 2020 Jul 28;10:1234. doi: 10.3389/fonc.2020.01234 (PMC7399223; doi:10.3389/fonc.2020.01234)
Supplement: Supplementary file 1 [file Data_Sheet_1.docx]

**Supplementary Table S1. The clinical information of the patients in Fig1.**

| Parameters | | Total | Patients treated with Intravesical Chemotherapy | | Patients treated with Neoadjuvant Chemotherapy | |
| --- | --- | --- | --- | --- | --- | --- |
| All patients | | 20 | 10 | | 10 | |
| Gender male | | 17 | 8 | | 9 | |
| Female | | 3 | 2 | | 1 | |
| Age at diagnosis, | |  | 39-85 | | 50-83 | |
| years old | |  |  |  |  |  |
| Smoking No | | 14 | 8 | | 6 | |
| Yes | | 6 | 2 | | 4 | |
| Tumor grade | |  |  | |  | |
| high | | 16 | 6 | | 10 | |
| low | | 4 | 4 | | 0 | |
| Drug | - | | | gemcitabine | | gemcitabine +cisplatin |

**Supplementary Table S2. The clinical information of the patients in Fig 6B.**

| Parameters | Total |
| --- | --- |
|  |  |
| All patients | 12 |
| Gender male | 10 |
| Female | 2 |
| Age (years) < 55 | 2 |
| ≥55 | 10 |
| Smoking No | 4 |
| Yes | 8 |
| Pathological T stage |  |
| Ta-T1 | 3 |
| T2-T4 | 9 |
| Pathological N stage |  |
| pNx or pN0 | 10 |
| pN1-2 | 2 |
| Tumor grade |  |
| high | 11 |
| low | 1 |

**Supplementary Table S3. The clinical information of the tissue microarray in Fig 6C-6F.**

| Parameters | Total | LSD1 expression | |
| --- | --- | --- | --- |
|  |  | Low (%) | High (%) |
| All patients | 107 | 36 (33.6) | 71 (66.4) |
| Gender male | 99 | 34 (34.3) | 65 (65.7) |
| Female | 8 | 2 (25.0) | 6 (75.0) |
| Age (years) < 55 | 19 | 8 (42.1) | 11 (57.9) |
| ≥55 | 88 | 28(31.8) | 60 (68.2) |
| Smoking No | 39 | 11(28.2) | 28(71.8) |
| Yes | 68 | 25(36.8) | 43(63.2) |
| Pathological T stage |  |  |  |
| Ta-T1 | 40 | 13(32.5) | 27(67.5) |
| T2-T4 | 67 | 23(34.3) | 44(65.7) |
| Pathological N stage |  |  |  |
| pNx or pN0 | 84 | 29(34.5) | 55(65.5) |
| pN1-2 | 23 | 7(30.4) | 16(69.6) |
| Tumor grade |  |  |  |
| high | 84 | 23(27.3) | 61(72.7) |
| low | 23 | 13(56.5) | 10(43.5) |

**Supplementary Table S4. Oligonucleotides used in this study**

| GenesID | Forward | | Reverse |
| --- | --- | --- | --- |
| Oligonucleotides for expression studies | | | |
| LSD1 | ACACCCCGCAAGAAAGAGC | | GACCCAGGCACGACAGTAG |
| LEF1 | AGAACACCCCGATGACGGGA | | GGCATCATTATGTACCCGGAAT |
| E-cadherin | CGAGAGCTACACGTTCACGG | | GGGTGTCGAGGGAAAAATAGG |
| N-cadherin | GCGGAGATCCTACTGGACGGT | | CCCTTGGCTAATGGCACTTGA |
| Vimentin | GACGCCATCAACACCGAGTT | | CTTTTGTCGTTGGTTAGCTGGT |
| Twist | GTCCGCAGTCTTACGAGGAG | | GCTTGAGGGTCTGAACTTGCT |
| Oligonucleotides for CHIP | | | |
| LEF1 | ACACACCCCAAAACCAAGAC | GAAGGAGGTGGTGATTGAGG | |

**
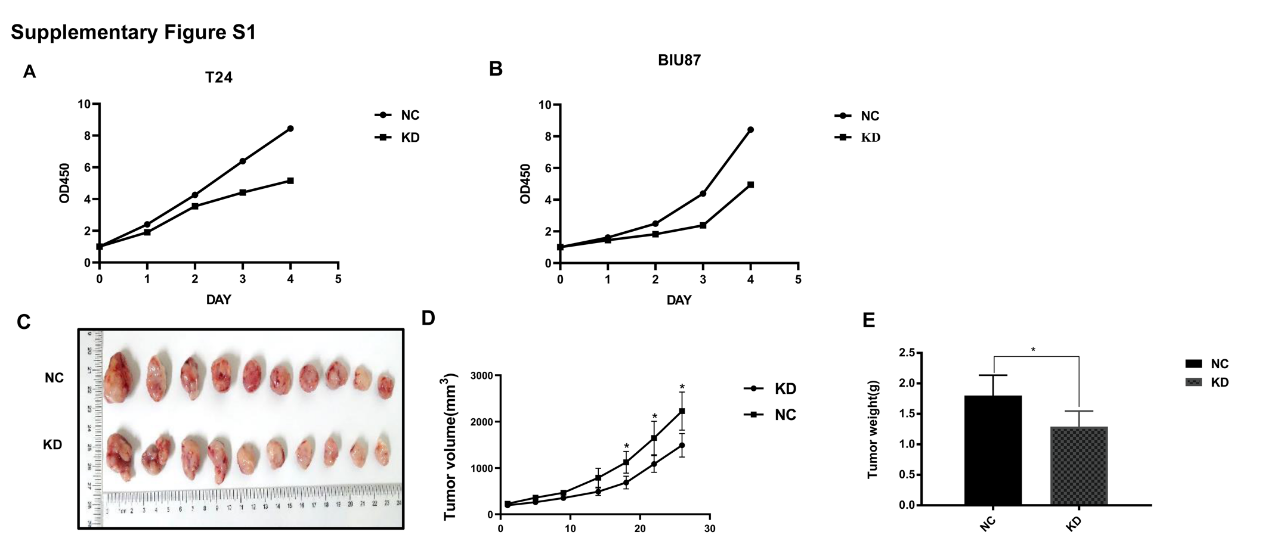
**

**Supplementary Figure S1: *LSD1 promotes BCa cell proliferation***

**(A-B)** T24 and BIU87 cells were seeded in 96-well plates with 1×10^3^ cells per well in growth media and treated with or without LSD1 knock down (2µM) for 7 days. Cell viabilities were estimated by CCK8 every other day. **(C-E)** Mice bearing xenografts of T24 cells with or without LSD1 knockdown were treated with GSK (2 µM), the tumors were collected and weighed. The data represent means ± S.D. The data represent means ± S.D. * P<0.05.


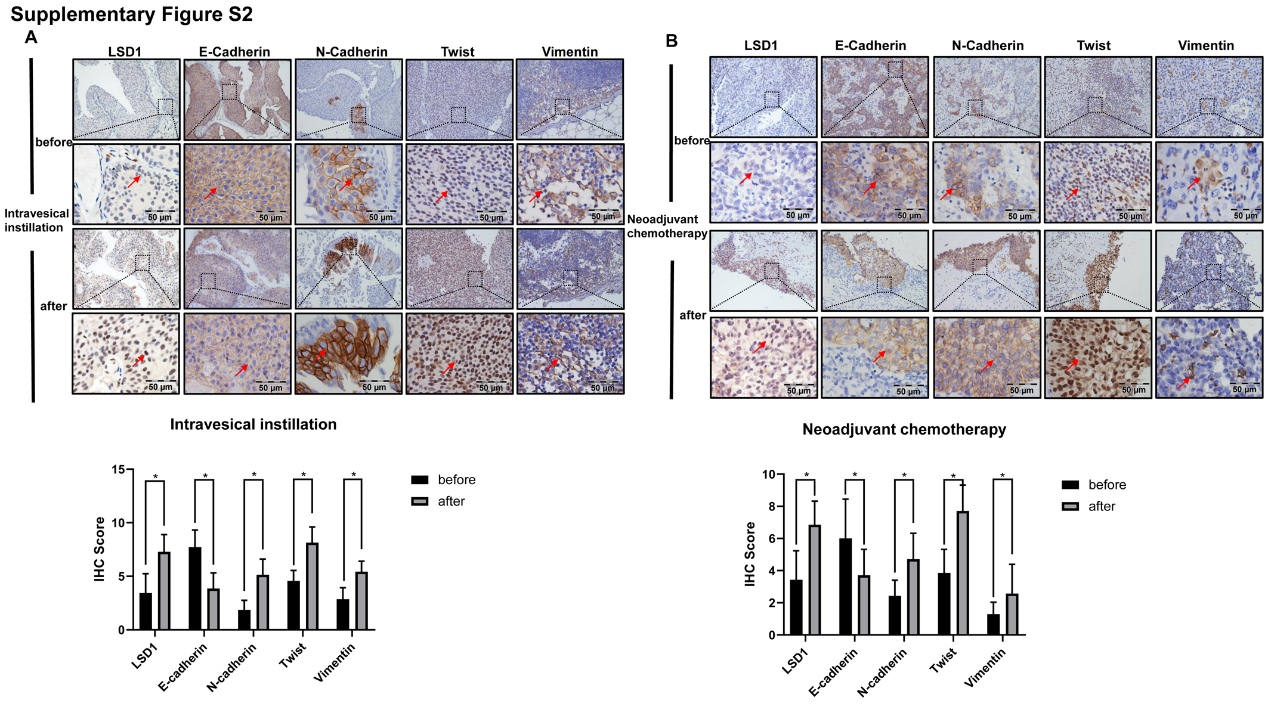


**Supplementary Figure S2: *The levels of LSD1 and EMT of BCa cells are enhanced by chemotherapy***

**(A)**Representative Immunostaining for LSD1 and EMT markers (E-cadherin, N-cadherin, Vimentin and Twist) in the same patient before and after intravesical instillation in NMIBC. **(B)** Representative immunostaining for above-mentioned markers before and after neoadjuvant therapy in MIBC. Magnifications: 20×, upper panels; Magnifications: 40×, bottom panels. * p < 0.05.
